# Detection of *Dirofilaria immitis* and other arthropod-borne filarioids by an HRM real-time qPCR, blood-concentrating techniques and a serological assay in dogs from Costa Rica

**DOI:** 10.1186/s13071-015-0783-8

**Published:** 2015-03-23

**Authors:** Alicia Rojas, Diana Rojas, Víctor M Montenegro, Gad Baneth

**Affiliations:** Departamento de Parasitología, Centro de Investigación en Enfermedades Tropicales, Facultad de Microbiología, Universidad de Costa Rica, P.O. Box 11501–2060, San José, Costa Rica; Laboratorio de Parasitología, Escuela de Medicina Veterinaria, Universidad Nacional, P.O. Box 86–3000, Heredia, Costa Rica; Koret School of Veterinary Medicine, Hebrew University of Jerusalem, P.O. Box 12, Rehovot, 76100 Israel

**Keywords:** *Dirofilaria immitis*, *Acanthocheilonema reconditum*, *Cercopithifilaria bainae*, Canine filariosis, PCR, Knott’s test, Costa Rica

## Abstract

**Background:**

Canine filarioids are important nematodes transmitted to dogs by arthropods. Diagnosis of canine filariosis is accomplished by the microscopic identification of microfilariae, serology or PCR for filarial-DNA. The aim of this study was to evaluate a molecular assay for the detection of canine filariae in dog blood, to compare its performance to other diagnostic techniques, and to determine the relationship between microfilarial concentration and infection with other vector-borne pathogens.

**Methods:**

Blood samples from 146 dogs from Costa Rica were subjected to the detection of canine filarioids by four different methods: the microhematocrit tube test (MCT), Knott’s modified test, serology and a high resolution melt and quantitative real-time PCR (HRM-qPCR). Co-infection with other vector-borne pathogens was also evaluated.

**Results:**

Fifteen percent of the dogs were positive to *Dirofilaria immitis* by at least one of the methods. The HRM-qPCR produced distinctive melting plots for the different filarial worms and revealed that 11.6% of dogs were infected with *Acanthocheilonema reconditum*. The latter assay had a limit of detection of 2.4x10^−4^ mf/μl and detected infections with lower microfilarial concentrations in comparison to the microscopic techniques and the serological assay. The MCT and Knott’s test only detected dogs with *D. immitis* microfilaremias above 0.7 mf/μl. Nevertheless, there was a strong correlation between the microfilarial concentration obtained by the Knott’s modified test and the HRM-qPCR (*r* = 0.906, *p* < 0.0001). Interestingly, one dog was found infected with *Cercopithifilaria bainae* infection. Moreover, no association was found between microfilaremia and co-infection and there was no significant difference in microfilarial concentration between dogs infected only with *D. immitis* and dogs co-infected with *Ehrlichia canis, Anaplasma platys* or *Babesia vogeli.*

**Conclusions:**

This is the first report of *A. reconditum* and *C. bainae* in Costa Rica and Central America. Among the evaluated diagnostic techniques, the HRM-qPCR showed the most sensitive and reliable performance in the detection of blood filaroids in comparison to the Knott’s modified test, the MCT test and a serological assay.

**Electronic supplementary material:**

The online version of this article (doi:10.1186/s13071-015-0783-8) contains supplementary material, which is available to authorized users.

## Background

Canine arthropod-borne filarioids include nematodes of the superfamily Filarioidea which are transmitted by arthropods such as mosquitos, fleas, lice and ticks [1]. *Dirofilaria immitis, D. repens, Acanthocheilonema reconditum, Onchocerca lupi* and *Thelazia callipaeda* are among the most important species that affect dogs*.* Animals infected with these parasites may remain asymptomatic or suffer from subcutaneous abnormalities, formation of nodules in subcutaneous tissues or life-threatening pathologies that include cardiovascular complications [2].

The distribution of canine filarioids depends on the presence of the vector, climate conditions (such as temperature, relative humidity and precipitation), density of human population and the presence of other canid populations that serve as reservoirs for these filarioids [3]. In the case of Costa Rica, *D. immitis* is the only canine filarioid reported to date. In 2009, a seroprevalence study of 84 owned dogs revealed that 2.3% were infected with heartworm [4]. In addition, seven cases of human dirofilarosis have been reported in Costa Rica since 1984 [5-9].

The diagnosis of canine filarosis in clinical laboratories can be accomplished by the identification of microfilariae, serology or PCR for filarial DNA from the dog’s blood. The gold standard of filarial detection has been the modified Knott’s test, which relies on the observer’s expertise and ability to morphologically identify microfilariae concentrated from the blood [10]. Serological diagnosis of *D. immits* is based on the detection of a female adult antigen, and has been applied for clinical purposes and in epidemiological studies [11]; however, it restricts detection only to *D. immitis*, disregarding other canine filarioids. Molecular detection techniques have been designed to detect different genetic loci that identify canine filarioids in general or certain species with high sensitivity and specificity [12-15]. Nevertheless, none of previous studies have compared the validity of the Knott’s test to all the other diagnostic methods included in this study.

The purpose of this study was to evaluate infection of canine filarioids by two blood-concentrating techniques (Knott’s modified test and the Microcapillary test), a serological assay and a novel quantitative HRM real-time qPCR; to compare the performance of the tests; and to determine the relationship between microfilarial concentration and co-infection with other vector-borne pathogens, demographic data and PCV values.

## Methods

### Animals and sample collection

One hundred and forty six blood samples from dogs were obtained from the Costa Rican regions of San Ramón, Alajuela (Costa Rica’s Central Valley, elevation 1060 m); Kéköldi, Limón (The Atlantic coast, elevation 169 m); Liberia, Guanacaste (Pacific coast, elevation 142 m) and Chomes, Puntarenas (Pacific Coast, elevation 8 m), during the rainy season (May to November) of 2012 as a part of a previous study [16]. The regions were chosen because they represented different geophysical and climate conditions. A questionnaire was filled for each animal with information regarding sex and age. Blood was obtained from the cephalic vein and collected in EDTA and serum tubes. The samples were transported at 4°C to the laboratory. After allowing blood to clot, sera were separated by centrifugation and stored at −20°C until further analysis. The packed cell volume (PCV) of each dog was measured by glass microcapillary centrifugation from EDTA blood samples. Dogs were divided into three groups according to their PCV value: group 1 (PCV: 7-24%), group 2 (PCV: 25-34%) and group 3 (PCV: 35-50%). The study was approved by the Inter-Institutional Committee for the Care and Use of Animals (CICUA), Universidad de Costa Rica.

### Microcapillary test (MCT)

EDTA blood samples were centrifuged in microhematocrit tubes and the buffy coat was analyzed for the presence of microfilariae by light microscopy at 100 and 400 magnifications. The number of microfilariae was recorded for each sample, as described elsewhere [17].

### Knott’s modified test

Knott’s modified test was performed with EDTA blood samples from dogs as described by Castillo and Guerrero [18] with the following modifications. Briefly, 0.5 ml of EDTA blood was added to 4.5 ml of 2% formalin, mixed by inversion and centrifuged at 3000 × g for 5 minutes. The volume of supernatant was measured for each sample and later discarded. The sediment was mixed with 35 μl of 0.1% methylene blue and 20 μl of this mixture were observed by a light microscope at 100× and 400× magnifications. No morphometric distinction was made between microfilariae of different species. The number of microfilariae per microliter (mf/μl) was calculated according to the following formula:

$$ mF/\mu l=\frac{\mu F\  observed\times \left\{\left[\left({V}_{blood}+{V}_{formalin}\right)-{V}_{supernatant}\right]+{V_{methylene}}_{blue}\right\}}{V_{sample}\times {V}_{blood}} $$

### Serological examination

The commercial kit VetScan® Canine Heartworm Rapid Test (Abaxis Inc, Union City, CA) was employed for the detection of *D. immitis*. This rapid assay detects circulating *D. immitis* female adult antigen in sera and the manufacturer declares a sensitivity and specificity of 98% and 100%, respectively [19]. The test was performed and its results were interpreted according to the manufacturer’s instructions.

### DNA extraction from dog samples

DNA from EDTA blood samples was extracted with a commercial kit (Illustra Blood Genomic Prep Mini Spin Kit, GE Healthcare, Buckinghamshire, UK), following the manufacturer’s instructions.

### Screening for filaroid-DNA with HRM real-time PCR

A high resolution melt (HRM) real-time PCR was performed using primers that target a partial sequence of the mitochondrial *12S* gene of filarioids of approximately 115 bp [15]. Primers (F5′-TTTAAACCGAAAAAATATTGACTGAC-3′ and R5′- AAAAACTAAACAATCATACATGTGCC-3′) were designed to detect *D. immitis, Brugia malayi* and *B. pahangi* [15] but they are also able to amplify the DNA of other filarial species. Three microliters of each DNA sample were diluted in a final volume of 20 μl with 10 μl of Maxima Hot Start PCR Master Mix (Thermo Fisher Scientific Inc., Surrey, UK), 4.4 μl sterile PCR grade water, 0.6 μl of SYTO-9 (Invitrogen, Carlsband, US) and 1 μl of each primer at 500 nM. The protocol was modified by performing an initial hold of 4 min at 95°C and 50 cycles of 5 s at 95°C, 15 s at 58°C and 10 s at 72°C. The melt curve was constructed from 60°C to 95°C with increments of 1°C/sec, followed by a hybridization step. An HRM curve was measured from 70 to 85°C at 0.1°C/sec. Reactions were performed with a Rotor Gene 6000™ cycler (Corbet, Sydney, AU). All runs included a non-template control (NTC) with PCR-grade water and DNA from a laboratory bred pathogen-free dog’s blood sample. As positive controls, DNA extracted from blood samples with *D. immitis* and *A. reconditum,* from heavily infected dogs from Puntarenas and Guanacaste, Costa Rica, respectively, were used and run in each reaction. Additionally, DNA from *D. repens-*positive blood samples from Israel were employed for the standardization of the assay. All positive amplicons obtained in the study were confirmed by sequencing (described below).

### Co-infection analysis

Specific PCR reactions for *D. immitis* and *A. reconditum* were performed to detect potential co-infection cases in the positive samples detected by the general filaroid HRM real-time PCR (described above). *D. repens-*detection was not tested due to reported absence of this filarioid in the Americas [20]. Accordingly, positive samples for *A. reconditum* were run in a HRM real-time PCR specific for *D. immitis*, and the positive samples for *D. immitis* were run in a HRM-real time PCR specific for *A. reconditum*.

*Dirofilaria immitis* detection was targeted using primers DI COI-F1 (5′- AGTGTAGAGGGTCAGCCTGAGTTA-3′) and DI COI-R1 (5′- ACAGGCACTGACAATACCAAT-3′) [12] at a concentration of 250 nM, which amplify a 200 bp fragment of the cytochrome oxidase (*cox1*) gene of *D. immitis*. The conditions consisted of an initial hold of 4 min at 95°C and 50 cycles of 15 sec at 95°C, 30 sec at 59°C and 5 sec at 72°C. The melt curve went from 60°C to 95°C with a raise of 1°C/1 sec, followed by a hybridization step from 90°C to 50°C. Finally, an HRM curve was performed from 70°C to 82°C, with an increment of 0.1°C/sec. Each run included a non-template control with PCR grade water, a negative control and a positive control of *D. immitis*.

The HRM real-time PCR for *A. reconditum-*DNA was carried out using primers AR COI-F1 (5′- AGTGTTGAGGGACAGCCAGAATTG-3′) and AR COI-R1 (5′-CCAAAACTGGAACAGACAAAACAAGC-3′) at a concentration of 500 nM, which amplify a 200 bp fragment of the cytochrome oxidase (*cox1*) gene of *A. reconditum*. The conditions consisted of an initial hold of 4 min at 95°C and 50 cycles of 15 sec at 95°C, 30 sec at 59°C and 5 sec at 72°C. The melt curve went from 60°C to 95°C with a raise of 1°C/1 sec, followed by a hybridization step from 90°C to 50°C. Finally, an HRM curve was performed from 70°C to 85°C, with an increment of 0.1°C/sec.

### Quantitative HRM real-time PCR (HRM-qPCR) for *D. immitis*

A standard curve for the absolute quantification of *D. immitis* by HRM-qPCR was developed. Accordingly, a serial dilution of the DNA extracted from the blood of a *D. immitis*-infected dog with known microfilariae concentration (14.33 *D. immitis* mf/μl of blood, determined twice by the Knott’s modified test) was used as standard points for the curve. This quantitative real-time PCR targets the mitochondrial *12S* gene of filarial species with conditions and reaction volumes as described above. Thus, three-fold serial dilutions of the DNA-positive control were prepared in sterile PCR grade water (Sigma, St. Louis, USA). The serial dilutions ranged from 1.4x10^1^ to 8.1x10^−6^ mf/μL. All the points of the standard curve (11 in total) were analyzed by triplicate. The standard curve was prepared with a logarithm of mf/μl versus the threshold cycle (Ct) values. The slope, intercept, efficiency and *R*^*2*^ values from this curve were obtained.

All the positive samples for *D. immitis* were quantified with the standard curve. The estimated microfilarial concentration (mf/μl) was calculated by the interpolation of the Ct value of each sample to the standard curve equation. In order to normalize the variations within and between PCR runs, a correction factor was calculated. The correction factor of each run was obtained with the division of the Ct of the standard point obtained in the standard curve and the Ct of the same point in each run for the sample analysis. Then, the Ct of each sample was corrected by the multiplication of the correction factor against each sample Ct value.

### DNA sequencing

Positive DNA amplicons were purified (EXO-Sap, New England Biolabs Inc., Ipswich, MA, USA) and subsequently sequenced using the BigDye Terminator cycle sequencing chemistry from Applied Biosystems ABI3700 DNA Analyzer, and the ABI’s Data Collection and Sequence Analysis software (ABI, Carlsbad, US). Samples were identified when the sequence of the amplicon indicated that the closest GenBank accession was at least 97% identical to the identified species. The data was analyzed with the Chromas Lite Version 2.01 software and compared to database available from GenBank using BLASTn 2.2.26 program (http://www.ncbi.nlm.nih.gov/BLAST).

### Statistical analysis

Infection rates (%) of canine filarioids were expressed with confidence intervals of 95%. To estimate the potential association between nominal variables, the Chi-square or Fisher’s exact tests were applied, according to the sample size. A two-tailed *T*-test was employed to evaluate differences between microfilarial concentrations in dogs infected only with *D. immitis* and those co-infected with *Babesia vogeli, Hepatozoon canis, Ehrlichia canis* or *Anaplasma platys* [16], and to evaluate the difference in microfilarial concentration and PCV values. A two-tailed Pearson correlation test and a linear regression test were performed to evaluate the correlation between the microfilarial concentration obtained by the Knott’s modified test and the HRM real-time qPCR. A paired two-tailed *T*-test was employed to compare the mean microfilarial concentration of *D. immitis* obtained by the Knott’s modified test and the HRM real-time qPCR. Additionally, Cohen’s kappa coefficient was calculated to determine the agreement in the detection of cases of *D. immitis*-infection between the four diagnostic tests employed. The statistical tests were analyzed under the hypothesis of null independence. Significance was determined with *p* <0.05. The Bonferroni correction was applied in cases where multiple comparisons were performed. Statistical analyses were performed using the IBM SPSS Statistics 20.0 software.

## Results

### Microcapillary test (MCT)

A total of 8.9% (13/146; 95% C.I.:5.0-14.4%) of the blood samples were positive to filarioids by the MCT (Additional file 1: Table S1). The MCT did not distinguish between microfilariae species. All positive dogs were from the region of Chomes, Puntarenas.

### Knott’s modified test

Seventeen percent (25/146, 95% C.I.: 11.4-24.2%) of the dogs were found to harbor microfilariae by this test. This test was not employed to distinguish between microfilariae species. All of the microfilariae were found in dogs from Chomes, Kéköldi and San Ramón (Additional file 1: Table S1). The average number of microfilariae ranged from 0.05 to 22.7 mf/μl. The dogs from Chomes presented the highest microfilaremia compared to the other two locations (Fisher’s exact test, *p <* 0.001).

### Serological assay

Eleven percent of the samples (16/146, 95% C.I.: 6.4-17.2%) were positive for *D. immitis* antigen (Additional file 1: Table S1). Additionally, two samples were classified as inconclusive according to the interpretation of the results as classified by the manufacturer.

### Molecular methods

**Figure 1:**
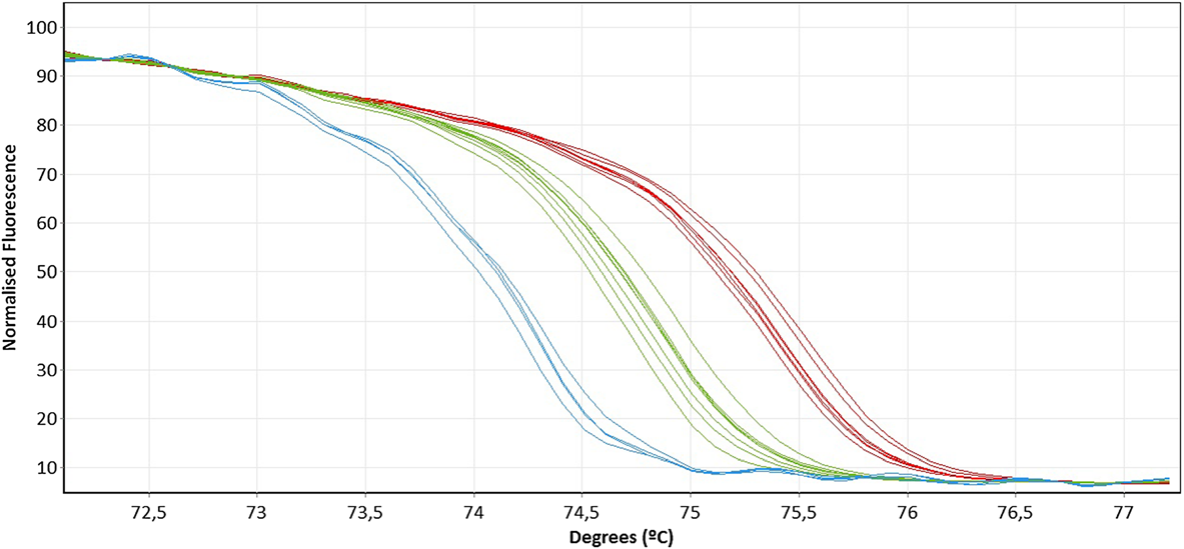
**HRM real-time qPCR analysis for the identification of** ***12S*** **rRNA gene of canine filarioids.** Normalized HRM curves of positive samples with *Acanthocheilonema reconditum* (blue) (Tm = 74.39 ± 0.03°C), *Dirofilaria immitis* (green) (Tm = 74.92 ± 0.04°C) and *D. repens* (red) (Tm = 75.54 ± 0.05°C).

The HRM real-time PCR screening revealed that 22.6% of the dogs (33/146, 95% C.I.: 16.1-30.2%) were positive for filarioid DNA. Of these, 51.5% (17/33, 95% C.I.: 33.5-69.2%) were identified as *D. immitis* and 48.5% (16/33. 95% C.I.: 30.8-66.5%) as *A. reconditum* according to their DNA sequences. Moreover, *A. reconditum* and *Dirofilaria* spp. produced clearly distinct HRM curves (Figure 1). The GenBank accession numbers with the closest match and identity percentages for *D. immitis* DNA sequences were FN391554.1 (97%) and HQ540423.1 (100%), and for *A. reconditum* JF461460.1 (97%).

No cases of dog co-infection with *D. immitis* and *A. reconditum* were revealed by the specific HRM real-time PCRs.

One dog with a positive result in the HRM real-time PCR screening for filarioids presented a low quality inconclusive sequence with the closest match to a dermal filarioid. Therefore, skin scrapes and conjunctival swabs of this dog obtained from a previous study [16] were submitted to the Dipartamento di Medicina Veterinaria, Università degli Studi di Bari, in Italy, for additional testing. PCR for the detection of the genes *12S* and *cox1* of *Cercopithifilaria* sp*.* was performed on these samples and revealed the presence of *Cercopithifilaria bainae* (100% identity to GenBank accession numbers JF461461 and JF461457 for *12S* and *cox1* genes, respectively).

### Quantification of *D. immitis* by the HRM real-time PCR

The standard curve for the quantification of *D. immitis* is shown in the Additional file 2: Figure S1. The curve had an efficiency of 96%, *R*^*2*^ = 0.985 and a limit of detection of 2.4×10^−4^ mf/μl. The microfilaremia of the dogs ranged from 6.6×10^−6^ to 34.2 mf/μl. Three dogs presented lower microfilaremia than the lowest concentration in the curve. Additionally, two dogs had higher microfilaremia than the highest point of the standard curve. The concentrations of these samples were calculated by the extrapolation of the curve assuming linearity, and, thus should be considered as estimated values. The assay’s detection limit was 2.4×10^−4^ mf/μl.

### Evaluation of method performance

Filaroids were detected in 24.0% (35/146, 95% C.I.: 17.3-31.7%) of the dogs by putting together results from all of the employed methods. The HRM real-time qPCR detected 94.3% (33/35, 95% C.I.: 80.8-99.3%) of the total positives, whereas the MCT, the Knott’s test and the serological assay detected 37.1% (13/35, 95% C.I.: 21.5-55.1%), 71.4% (25/35, 95% C.I.: 53.7-85.7%) and 45.7% (16/35, 95% C.I.: 28.8/63.4%), respectively.

*Dirofilaria immitis* was identified by the HRM real-time PCR and the serological assay in 15.10% (22/146, 95% C.I.: 9.7-22.0%) of the samples. The HRM real-time PCR detected 77.3% (17/22, 95% C.I.: 54.6-92.2%) and the serological assay 72.7% (16/22, 95% C.I.: 49.8-89.3) of the *D. immitis*-positive dogs (Table 1). Five samples were detected only by the serological assay and 6 only by the HRM real-time PCR. There was a moderate statistical agreement in the detection of *D. immitis* by the HRM real-time PCR and the Knott’s and microcapillary tests (all κ > 0.522, all *p* < 0.005), and perfect agreement between Knott’s test and the microcapillary test (κ = 0.91, *p* < 0.0001). However, there was no agreement in the detection of dirofilariosis cases by the serological assay and the HRM real-time PCR, Knott’s and microcapillary tests (all κ < −0.09, all *p* > 0.12).

**Table 1:** **Comparative detection of** ***Dirofilaria immitis*** **by different diagnostic assays**

**Diagnostic technique**	**Number of positive dogs**	**% of all dogs positive for** ***Dirofilaria immitis*** **by PCR or serology**
HRM real-time PCR with sequencing and serology	22	100%
HRM-PCR with sequencing	17	77%
Serology	16	73%
KMT	13	59%
MCT	12	54%

Dogs positive by HRM real-time PCR and DNA sequencing or by specific serology were considered as truly positive. The table compares how many of these truly positive dogs were also positive by the Knott’s modified test (KMT) and the microcapillary test (MCT).

**Figure 2:**
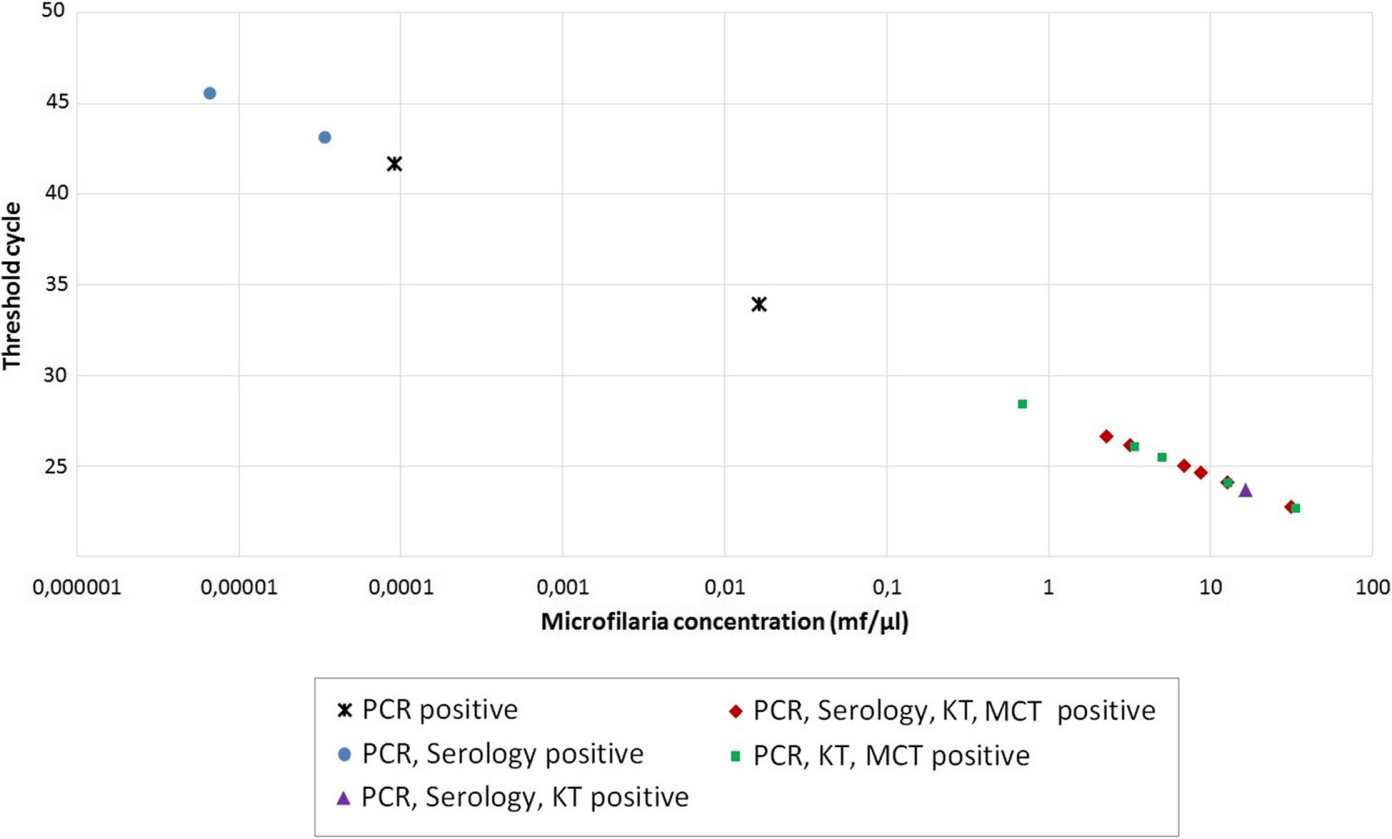
**Comparison of the HRM real-time qPCR, microscopic and serological methods in** ***D. immitis-*** **detection.** Each point of the curve corresponds to a sample positive for *D. immitis* according to the HRM real-time qPCR and/or the microcapillary test (MCT), Knott’s test (KT) and a serological assay. The concentration of mf/μl was obtained by interpolation to the standard curve. The MCT and KT detected only samples with concentrations higher than 0.7 mf/μl as shown in the high microfilaremic section of the graph.

**Figure 3:**
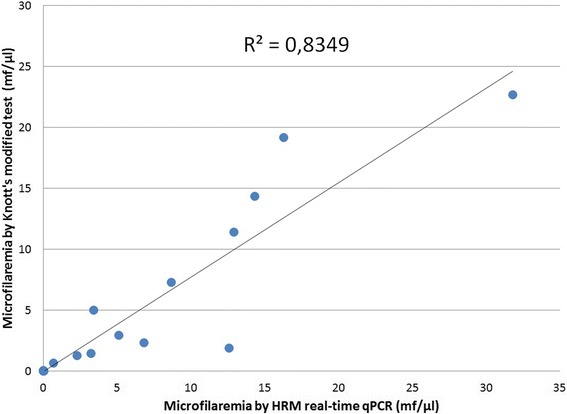
**Correlation between microfilarial concentrations obtained by HRM real-time qPCR for** ***D. immitis*** **and the Knott’s test.** The coefficient of linear regression is shown in the graph. Each point corresponds to a different dog blood sample.

The quantification of the microfilaremia level by the HRM-qPCR allowed the comparison of positive samples detected by other tests as well. Accordingly, the MCT and Knott’s test only detected dogs with microfilaremias above 0.7 mf/μl revealed by the HRM-qPCR as positive and missed 4 dogs with lower concentrations of microfilariae (Figure 2). However, three samples, detected as positive for microfilariae by the microscopic methods, were found negative by this molecular assay. The serological assay detected cases of dirofilarosis among samples of all microfilariae concentrations (Figure 2), and 5 samples were only found serologically positive and negative by the techniques dependent on detection of microfilaremia (MCT, Knott’s test and HRM-qPCR). The mean value of microfilariae/μl obtained by either the HRM-qPCR (6.94 ± 8.5 mf/μl) and/or the Knott’s test (5.32 ± 7.22 mf/μl) did not vary significantly (two-tailed Paired *T*-test, d.f.: 15, all *p* = 0.075). Moreover, there was a strong positive correlation between the microfilarial concentration obtained by the Knott’s modified test and the HRM real-time qPCR (two-tailed Pearson correlation test, r = 0.906, *p* < 0.0001) (Figure 3).

The real-time PCR identified filarioids that the other assays could not detect. *Acanthocheilonema reconditum* was correctly identified only by the real-time PCR and confirmed by sequencing in 11.0% (16/146, 95% C.I.: 6.4-17.2%) of the samples. In this regard, the serological assay, did not present cross reaction with this filarial sp.

### Co-infection with vector-borne hemopathogens and *D. immitis*

Sixty five percent (11/17, 95% C.I.: 38.3-85.8%) and 19% (3/16, 95% C.I.: 4.1-45.6%) of the dogs with molecularly detected-*D. immitis* (Table 2) and *A. reconditum,* respectively, were co-infected with protozoal or bacterial vector-borne pathogens such as *Babesia vogeli, Ehrlichia canis* and *Anaplasma platys* detected in our previous study [16]. There was no difference in microfilarial concentration between dogs with single infection with *D. immitis* (mean concentration: 13.1 ± 16.2 mf/μl); and co-infected with the other hemopathogens (mean concentration: 6.7 ± 5.6 mf/μl) (two-tailed *T*-test, T = 0.58, d.f. = 15, *p =* 0.238). Noteworthy, the only dog in the study that presented co-infection with three pathogens (*B. vogeli and A. platys*), had the lowest microfilaremia (6.6×10^−6^ mf/μl).

**Table 2:** ***Dirofilaria immitis*** **-microfilarial concentration and co-infection with other vector-borne hemopathogens in dogs from Costa Rica**

**Pathogens detected**	**Number of dogs infected by the detected pathogens (% of total number of dogs)**	Mean microfilaremia numbers ± standard deviation (mf/μl)*
*Dirofilaria immitis* only	6 (4.1%)	13.1 ± 16.2
*D. immitis* and *Anaplasma platys*	1 (0.7%)	16.3
*D. immitis* and *Ehrlichia canis*	9 (6.1%)	6.3 ± 4.7
*D. immitis, A. platys* and *Babesia vogeli*	1 (0.7%)	6.6×10^−6^
Total	17 (11.6%)	9.0 ± 10.6

The concentrations were calculated by quantitative HRM real-time qPCR.

*No statistical differences were found.

### Association of location, age, sex and PCV values with detection of filarioids

The presence of filarioids according to the detection by the HRM real-time PCR, varied in regards to the location, sex, age and PCV value of the dogs (Additional file 3: Table S2). The distribution of *D. immitis* and *A. reconditum* was significantly higher in Chomes and Kéköldi, respectively, than in the other sampled regions (Chi-square test *p*< 0.0001 for each location). With regard to age, 82% (14/17, 95% C.I.: 56.6-96.2%) of the cases with *D. immitis* occurred in dogs younger than 4 years, and 50% (8/16, 95% C.I.: 24.6-75.3%) of the dogs with *A. reconditum* were younger than 1 year. Infection with these filarioids was observed in more males (29.5%; C.I. 95%: 18.6-39.5%) than females (17.6%; C.I. 95%: 95–28.8%). However, no significant differences were found between filarioid-infection and sex or age of the dogs (Chi-square test, *p* = 0.132). Additionally, there was no significant difference between the PCV values of dogs with *D. immitis* or *A. reconditum* and the values of dogs negative for filarioids (two-tailed *T*-test *p* = 0.36 and *p =* 0.26, respectively).

## Discussion

Canine filarioids are arthropod-borne pathogens that cause severe disease to dogs and potentially also to humans. The wide distribution of these parasites is attributed to the adaptation of their vectors to their final hosts and the environment, as well as to climate changes [1]. This study describes the presence of *A. reconditum* and *C. bainae* in dogs from Costa Rica and Central America from the first time. Moreover, it compared the performance of three different methods employed currently in clinical practice and a novel HRM real-time qPCR for the detection of *D. immitis.*

*Dirofilaria immitis* was detected in 15% of the dogs sampled from Costa Rica by the combination of the HRM real-time PCR and a serological assay. The prevalence of *D. immitis* found in this study is higher than the 2.3% obtained in a previous serologic study from Costa Rica [4]. The higher prevalence of infection found may be explained by the use of a combination of detection techniques including molecular and serological assays, and also by sampling regions of Costa Rica with a potentially higher abundance of this nematode. We found that the coastal region of Chomes is endemic for the parasite since 88% of the cases were from this area and those dogs also had the highest microfilaremias. This finding is in agreement with the increased distribution of this filarioid in shorelines [21]. A study performed on convenience samples of dogs from neighboring Nicaragua did not detect *D. immitis* by PCR [22], however, serosurveys from the Caribbean and South America have described prevalence rates of infection that reach 74% [20]. No other *Dirofilaria* spp. were detected by our molecular assay, even though microfilariae resembling *D. repens* were reported recently in dogs from Chile [2].

*Acanthocheilonema reconditum* was detected in 11% of the sampled dogs. Prevalence studies in the Americas have reported rates of infection that range from 0.1% to 22% in the United States [23] and Brazil [24], respectively. The high prevalence of this filarioid in our study may be due to the widespread parasitism of *A. reconditum*’s intermediate hosts (e.g. fleas and lice) among dog populations. Despite the fact that the pathogenicity of *A. reconditum* is low compared to other filarioids [25], the occurrence of this parasite should be highlighted since it constitutes an important differential diagnosis for *D. immitis* in studies of dogs employing morphological detection techniques.

The HRM real-time qPCR performed in the present study successfully quantified *D. immitis*-microfilariae and distinguished filarioids that were not detected by the other employed assays. Moreover, there was a strong correlation between the microfilarial concentration obtained by Knott’s modified test and the current PCR (Figure 3). This can be explained by the use of a positive control quantified by Knott’s test for preparing the standard curve of the HRM-qPCR, as done in other qPCR protocols for detecting parasites [26]. These results show that, although the quantification of microfilariae by the assays were mostly similar, the qPCR had the advantage of detecting positive samples with lower microfilarial concentrations (Figure 2). The molecular assay employed herein was able to detect cases with very low microfilaremia, which may occur during initial microfilaremia or following incomplete treatment [27].

A previously reported duplex quantitative real-time PCR for the detection of *D. immitis* and *D. repens* found a lower limit of detection (8.0×10^−6^ mf/μl) than the present assay (2.4×10^−4^ mf/μl) [14]. Nevertheless, the present method has the advantage of detecting other filarioids with a single pair of primers and separating them based on their HRM-curves, which makes it less laborious in the screening of large numbers of dogs. A limitation of our method was the use of a positive sample as the starting point of the standard curve. The latter required the extrapolation of microfilaremia values above the curve. Although challenging, a potential solution to this limitation is the isolation, quantification and DNA-extraction of higher number of microfilariae obtained from an *in vitro* culture [28].

The microscopic assays, i.e. the MCT test and Knott’s modified method, were useful in detecting more than half of the infected dogs with filarioids. The difficulty in the identification presented in this study relies on the observation of only one microfilaria in more than 40% of the preparations and in the epidemiological bias of being in a previously unknown *A. reconditum* region. In clinical practice, both microscopic methods depend on the observer’s expertise to morphologically identify and classify microfilariae [10]. Additionally, microscopic methods are known to have lower sensitivity for detection of microfilariae compared to molecular tools, as demonstrated in the present study, which makes the diagnosis of cases with low parasite burdens or dogs exposed to parasiticides more difficult [29-31]. The fact that the MCT detected mainly dogs with a high microfilaremia (Figure 2) could be due to the small amount of blood employed for this test. On the other hand, the Knott’s test detected similar microfilarial concentration as the HRM-qPCR (Figure 3), but failed in the detection of four low-concentration positive samples (Figure 2). Moreover, three samples were detected as positive only by either MCT or Knott’s test (HRM real-time PCR negative) possibly due to the presence of PCR-inhibitors, low DNA extraction-yield or missidentification of filarioids. The present study highlights the importance of proper identification of different filarial species especially in samples with low concentrations of microfilariae, and emphasizes the importance of the application of more than one screening technique for epidemiological studies.

Serological tests are the preferred method to diagnose *D. immitis* infection in clinical practice due to their high sensitivity and simplicity [1]. Moreover, this assay detected *D. immitis* antigenemia in five dogs which were molecular and microscopically-negative. The latter are probably associated with occult infection in amicrofilaremic dogs as previously described [32]. The negative serological results in microfilarial-positive dogs may be due to a low female burden or previous adulticidal treatment [33].

The agreement in the detection of *D. immitis-*cases in the HRM real-time PCR, Knott’s modified test and the microcapillary test relies in the fact that these three assays detect circulating microfilariae. On the contrary, the serological assay did not statistically agree with molecular and microscopic methods since it detects circulating antigens present also in occult infection [31].

The majority of the dogs with *D. immitis* (65%) were co-infected with other vector-borne pathogens such as *E. canis, A. platys* and *B. vogeli*. This situation may worsen the dog’s clinical manifestations and complicate the diagnosis and treatment [34,35]. However, in our study co-infection was not found to alter the burden of infection of *D. immitis* as manifested by the microfilarial concentrations.

*Cercopithifilaria bainae*-infection was a surprising finding. This filarioid was first described from Brazil [36] and has since been reported in clinical cases from Italy [37], Romania [38] and Portugal [39]; and in ticks from Italy, Spain, Portugal, Greece, Brazil, Australia, Malaysia, South Africa and Pakistan [40]. Our study constitutes the first report of this nematode in Costa Rica and Central America. The intermediate host of this nematode, the tick *Rhipicephalus sanguineus* [41], was found in a third of the dogs included in this study [16]. Therefore, the screening of additional skin samples from other dogs in Costa Rica could better describe the real prevalence of *C. bainae* in this country.

## Conclusions

The present study molecularly detected *D. immitis, A. reconditum* and *C. bainae* in dogs from Costa Rica. The latter two were detected for the first time in Costa Rica and Central America. Among the employed techniques to detect filarioids, the HRM real-time qPCR was the most sensitive and had the advantage of detecting and accurately discriminating the filarial species found in the dog’s populations, in comparison with the Knott’s test, microcapillary test and a serological assay. Therefore, the implementation of molecular techniques in the diagnosis of canine filarioids in the clinical practice should be recommended.

## Additional files

Additional file 1: Table S1. Arthropod-borne helminth detection in dogs from Costa Rica according to diagnostic method and sampling location. Additional file 2: Figure S1. Standard curve for the quantification of *Dirofilaria immitis* by an HRM real-time qPCR. The equation of the curve and R2 are shown in the graph. Additional file 3: Table S2. Canine filarioids distribution in Costa Rica according to demographic and clinical data. Demographic data of dogs include sampling location, sex and age. Packed cell volume (PCV) values are shown as percentages.
