# Supplementary material for: Detection of Dirofilaria immitis and other arthropod-borne filarioids by an HRM real-time qPCR, blood-concentrating techniques and a serological assay in dogs from Costa Rica
Source: Parasit Vectors. 2015 Mar 23;8:170. doi: 10.1186/s13071-015-0783-8 (PMC4377020; doi:10.1186/s13071-015-0783-8)
Supplement: Additional file 1: Table S1. — Arthropod-borne helminth detection in dogs from Costa Rica according to diagnostic method and sampling location. [file 13071_2015_783_MOESM1_ESM.docx]

Additional file 1. **Arthropod-borne helminth detection in dogs from Costa Rica according to diagnostic method and sampling location**.

|  | Total number of dogs with a positive result for each test per region (% of the total dogs infected with the filarioid) | | | |  |  |
| --- | --- | --- | --- | --- | --- | --- |
| Assay | Chomes (n=38) | Liberia (n=36) | San Ramón (n=30) | Kéköldi (n=42) | Total number of positive dogs result^*^ | Filaroids reported by each assay |
| Microcapillary test (MCT) | 13 (100%) | 0 | 0 | 0 | 13 (8.9%) | N.D. |
| Knott’s modified test | 13 (52%) | 0 | 2 (8%) | 10 (40%) | 25 (17.0%) | N.D. |
| Serological assay | 14 (88%) | 0 | 1 (6%) | 1 (6%) | 16 (11.0%) | *Dirofilaria immitis* |
| HRM real-time PCR for filarioids | 15 (88%) | 1 (6%) | 0 | 1 (6%) | 17 (12%) | *Dirofilaria immitis* |
|  | 0 | 1 (6%) | 3 (19%) | 12 (75%) | 16 (11%) | *Acanthocheilonema. reconditum* |

*% of total number of dogs, n=146.

N.D.: not determined by this method.
