# Supplementary figures and images for: Detection of Dirofilaria immitis and other arthropod-borne filarioids by an HRM real-time qPCR, blood-concentrating techniques and a serological assay in dogs from Costa Rica
Source: Parasit Vectors. 2015 Mar 23;8:170. doi: 10.1186/s13071-015-0783-8 (PMC4377020; doi:10.1186/s13071-015-0783-8)

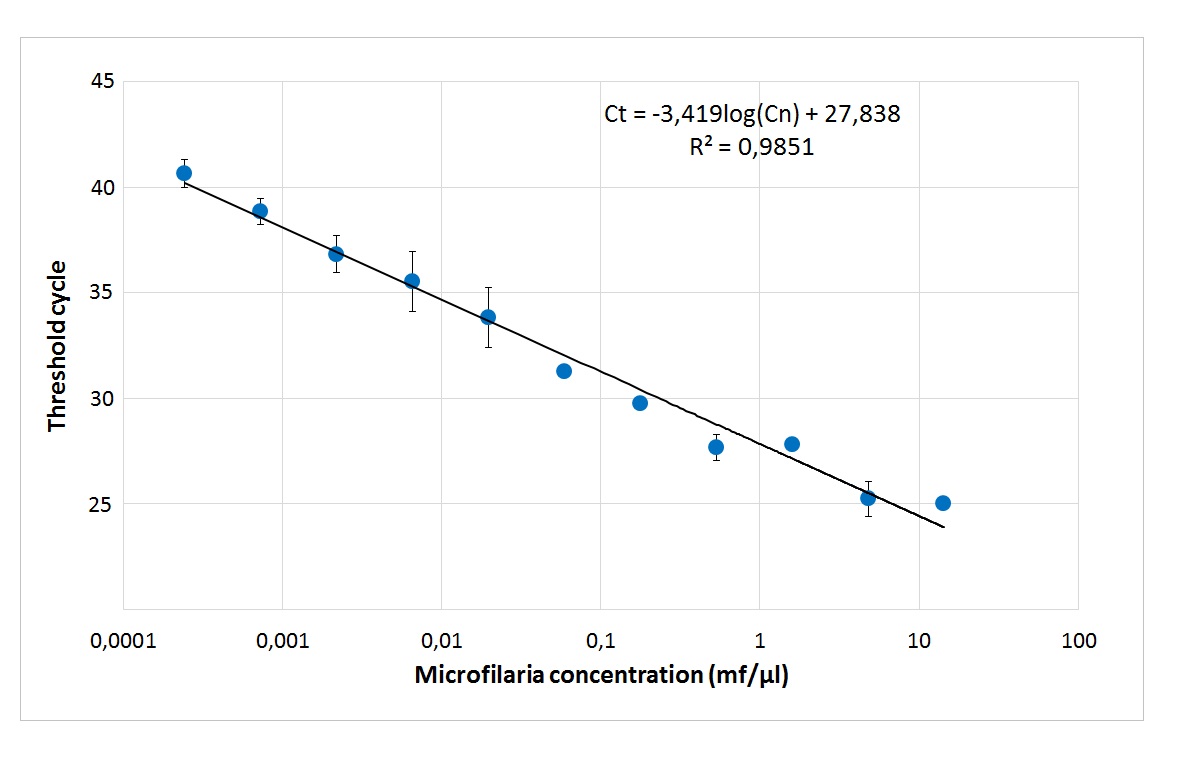

Supplement: Additional file 2: Figure S1. — Standard curve for the quantification of Dirofilaria immitis by an HRM real-time qPCR. The equation of the curve and R2 are shown in the graph. [file 13071_2015_783_MOESM2_ESM.jpg]
