# Supplementary material for: Detection of Dirofilaria immitis and other arthropod-borne filarioids by an HRM real-time qPCR, blood-concentrating techniques and a serological assay in dogs from Costa Rica
Source: Parasit Vectors. 2015 Mar 23;8:170. doi: 10.1186/s13071-015-0783-8 (PMC4377020; doi:10.1186/s13071-015-0783-8)
Supplement: Additional file 3: Table S2. — Canine filarioids distribution in Costa Rica according to demographic and clinical data. Demographic data of dogs include sampling location, sex and age. Packed cell volume (PCV) values are shown as percentages. [file 13071_2015_783_MOESM3_ESM.docx]

Additional file 3. **Canine filarioids distribution in Costa Rica according to demographic and clinical data.** Demographic data of dogs include sampling location, sex and age. Packed cell volume (PCV) values are shown as percentages.

|  | Number of dogs with *Dirofilaria immitis* infection detected by PCR | Number of dogs with *Acanthocheilonema reconditum* infection detected by PCR | Number of dogs not found infected by PCR | Total number of dogs |
| --- | --- | --- | --- | --- |
| **Sampling location** |  |  |  |  |
| Chomes, Puntarenas | 15 | 0 | 23 | 38 |
| Liberia, Guanacaste | 1 | 1 | 34 | 36 |
| Kékoldi, Limón | 1 | 12 | 29 | 42 |
| San Ramón, Alajuela | 0 | 3 | 27 | 30 |
| TOTAL | 17 | 16 | 113 | 146 |
| **Gender** |  |  |  |  |
| Female | 6 | 6 | 56 | 68 |
| Male | 11 | 10 | 57 | 78 |
| TOTAL | 17 | 16 | 113 | 146 |
| **Age (years)** |  |  |  |  |
| 0-1 | 2 | 8 | 39 | 49 |
| 1-4 | 12 | 2 | 48 | 62 |
| 5-7 | 3 | 3 | 16 | 22 |
| 8-12 | 0 | 3 | 10 | 13 |
| TOTAL | 17 | 16 | 113 | 146 |
| **PCV values^a^** |  |  |  |  |
| 7-24% | 6 | 2 | 17 | 25 |
| 25-34% | 6 | 10 | 49 | 65 |
| 35-50% | 5 | 4 | 46 | 55 |
| TOTAL | 17 | 16 | 112 | 145 |

^a^PCV could not be determined in one sample
